# Strangulated Hernia in a Child Five Months Old; Operation; Recovery

**Published:** 1852-11

**Authors:** 


*Strangulated Hernia in a Child five months old; Operation; recovery.* Under the care of Mr. Erichsen.—The following particulars were obtained from the notes of Mr. Turle, Mr. Erichsen's house-surgeon:

Arthur C——, aged five months and a half, was admitted April 12, 1852, under the care of Mr. Erichsen. It appears that when the boy was three weeks old, a hernial tumor appeared in the right groin; it could be easily reduced, and would only come down occasionally—viz: about once a week. The tumor then used to remain apparent for several hours, and afterwards ascend spontaneously into the abdominal cavity. When the child was four months old a truss was applied, but the apparatus proved ineffectual, as the tumor appeared again on the following day by the side of the pad. The truss seemed also to cause considerable pain, and the mother, therefore, brought the child to the hospital.

The little patient was found, on examination, to be affected with oblique inguinal hernia of the right side; the intestine had descended into the scrotum, and was greatly distending it. There were, however, no symptoms of strangulation at the time; some attempts were made to reduce the tumor, but these having failed, the mother was desired to leave off the truss and to bring the child immediately she perceived any unpleasant symptoms.

Six weeks after this (April 12, 1852) the mother applied to Mr. Erichsen again, as alarming signs of strangulation had manifested themselves. The tumor, which had remained unreduced, was now large and tense, its neck appearing to be tightly constricted by the external abdominal ring. In following the intestine down into the scrotum, it was remarked that the latter was not only distended by displaced intestine, but likewise by fluid secreted in the cavity of the tunica vaginalis, so that the child was suffering both from strangulated inguinal hernia and hydrocele. Constipation and vomiting had been existing for some time, the surface was cold, the face pale and drawn, and the patient evidently in a very precarious state.

The mother, having been told of the dangerous condition of her child, readily consented to the performance of the operation, and Mr. Erichsen proceeded, as soon as the child was narcotized by chloroform. The integuments were divided over the neck of the tumor, and the several layers of cellular tissue and faciæ having been carefully slit open, the sac was fairly exposed. The constriction was now found to be exerted by the external abdominal ring, and a few transverse fibres of the cremaster muscle; a curved director was passed beneath these parts, the strangulating structures were divided by an incision directly upwards, and the protruded intestine returned with facility without the peritoneal sac having been opened. The edges of the wound were approximated with sutures, and the whole secured by a compress and a double-headed roller.

Three hours after the operation a copious liquid motion was passed, the child being perfectly quiet and composed. On the following day he was in a very satisfactory state, and the wound looked very healthy. From this time the little patient progressed most favorably; he had no bad symptoms whatever, became cheerful, and improved much in appearance. The bowels were open daily, and the child left the hospital, the wound being quite cicatrized, eighteen days after admission.

It will be perceived that the taxis was not tried at all in this case, when the symptoms were fully established; and that this cautious conduct has a marked and beneficial influence on the issue, is clearly seen by the success here obtained. We cannot help noticing, in reviewing this case, the baneful effects of the common spring truss in infants; nor can it be otherwise, for it is next to impossible that the pad should exactly compress the ring with infants at the breast. It is equally difficult to regulate the spring in such a manner as to prevent the truss from slipping, without using an amount of pressure which must of necessity be hurtful to the child. Nothing will answer in such cases but elastic belts, which yield without becoming displaced when the infant cries; the belt being supplied with an air-pad, as suggested by M. Bourjeaurd, which shall be gently lodged upon the ring, and be made softer or harder, by means of the stop-cock, as occasion may require.—*London Lancet.*

